# Genome-wide analyses of the relict gull (*Larus relictus*): insights and evolutionary implications

**DOI:** 10.1186/s12864-021-07616-z

**Published:** 2021-04-29

**Authors:** Chao Yang, Xuejuan Li, Qingxiong Wang, Hao Yuan, Yuan Huang, Hong Xiao

**Affiliations:** 1 College of Life Sciences, Shaanxi Normal University, Xi’an, 710062 China; 2 Shaanxi Institute of Zoology, Xi’an, 710032 China

**Keywords:** Whole-genome, PacBio sequencing, *Larus relictus*, Habitat loss, Population fragmentation

## Abstract

**Background:**

The relict gull (*Larus relictus*), was classified as vulnerable on the IUCN Red List and is a first-class national protected bird in China. Genomic resources for *L. relictus* are lacking, which limits the study of its evolution and its conservation.

**Results:**

In this study, based on the Illumina and PacBio sequencing platforms, we successfully assembled the genome of *L. relictus*, one of the few known reference genomes in genus *Larus*. The size of the final assembled genome was 1.21 Gb, with a contig N50 of 8.11 Mb. A total of 18,454 genes were predicted from the assembly results, with 16,967 (91.94%) of these genes annotated. The genome contained 92.52 Mb of repeat sequence, accounting for 7.63% of the assembly. A phylogenetic tree was constructed using 4902 single-copy orthologous genes, which showed *L. relictus* had closest relative of *L. smithsonianus*, with divergence time of 14.7 Mya estimated between of them. PSMC analyses indicated that *L. relictus* had been undergoing a long-term population decline during 0.01-0.1 Mya with a small effective population size fom 8800 to 2200 individuals.

**Conclusions:**

This genome will be a valuable genomic resource for a range of genomic and conservation studies of *L. relictus* and will help to establish a foundation for further studies investigating whether the breeding population is a complex population. As the species is threatened by habitat loss and fragmentation, actions to protect *L. relictus* are suggested to alleviate the fragmentation of breeding populations.

**Supplementary Information:**

The online version contains supplementary material available at 10.1186/s12864-021-07616-z.

## Background

The relict gull (*Larus relictus*) (Charadriiformes, Laridae, *Larus*), a middle-sized gull with a black-coloured head, had been known for nearly 50 years before it was regarded as a unique species [1]. It is classified as vulnerable (VU) on the IUCN Red List and is a first-class national protected bird in China. Its population size has been estimated at 10,000–19,999 (BirdLife International, 2020), and the vast majority of *L. relictus* (90%) reside in Hongjian Nur with very low genetic diversity [2]. Their main wintering place is situated on the west coast of the Bohai Sea [3]. A small number of winter migratory individuals have been sighted in Hong Kong [4]. Therefore, the main threats to *L. relictus* are lake shrinkage on breeding grounds and at stopover sites, as well as the loss of intertidal flats on wintering grounds [5]. A novel data-driven habitat suitability ranking approach for *L. relictus* using remote sensing and GIS indicated that three threat factors, road networks, developed buildings and vegetation, affect suitable habitat for this species most severely [6].

On the whole-genome level, DNA sequencing technology is usually used to characterize genetic variation and acquire comprehensive molecular characterizations [7]. At present, only limited genetic information, in the form of mitochondrial markers and inferred population structure, is available for *L. relictus* [2, 8–10]. However, no genome has been published for *L. relictus* which limits our understanding about the molecular mechanisms of evolutionary and genetic processes.

High-throughput sequencing technology has notably reduced sequencing costs [11] and marked the start of a new era of genomic studies [12]. Among them, long-read sequencing technologies such as Pacific Biosciences (PacBio) [13] can produce average read lengths of over 10,000 bp [12]. PacBio technology has been used to obtain high-quality genome assemblies for several avian species, such as *Gallus gallus* (Galliformes) [14] and *Malurus cyaneus* (Passeriformes) [15].

In this study, the first contig-level genome of *L. relictus* was constructed using both Illumina HiSeq and PacBio sequencing platforms. We assessed various genomic characteristics and performed comparative analyses. These genomic data will facilitate population studies of *L. relictus* and support the comprehensive protection of this vulnerable avian species.

## Results

### Genome sequencing and assembly

Approximately 106.29 Gb of raw sequencing data were obtained using the Illumina HiSeq platform, including three 250-bp insert libraries and two 350-bp insert libraries (Table S1). The sequencing depth was 87.85X. We used the PacBio sequencing platform with three 20-Kb libraries to obtain long reads for assembling the genome and retained approximately 30.50 Gb raw data. The sequencing depth was 25.42X. After filtering out low-quality and short-length reads, the read N50 and mean read length were 12,712 bp and 8418 bp, respectively (Table S2, S3). Finally, a 1.21 Gb assembly with a contig N50 of approximately 8.11 Mb was obtained for *L. relictus*, with a GC content of approximately 43.11%. The genome consisted of 1313 contigs, with the longest contig being approximately 29.7 Mb long (Table S4).

Approximately 99.96–99.97% of the cleand Illumina reads could be mapped to the contigs, with 93.33–93.77% properly mapped reads (Table S5). The CEGMA v2.5 analysis identified 416 core eukaryotic genes (CEGs), accounting for 90.83% of all 458 CEGs, and 175 CEGs (70.56%) could be detected with homology to the 248 highly conserved CEGs (Table S6). In addition, 4555 (92.7%) of the 4915 highly conserved Aves orthologues from BUSCO v3.0.2 were identified in the assembly (Table S7). These results show that the assembled *L. relictus* genome sequence was complete and had a low error rate.

### Genome annotation

The consensus gene set included a total of 18,454 genes were predicted by three different strategies (Methods section for details) (Table S8). The average gene length, exon length, and intron length were 20,749.08 bp, 164.24 bp, and 1996.77 bp, respectively. The final prediction results revealed 17,452 (94.57%) supported by homology-based and RNA-seq-based methods (Fig. S1), which showed a good gene prediction efficiency compared to gene annotations of genomes in five known species of Laridae, human and *G. gallus* (Table S9) [16, 17]. A total of 16,967 (91.94%) predicted genes in the *L. relictus* genome were annotated and functionally classified by the Gene Ontology (GO) [18], Kyoto Encyclopedia of Genes and Genomes (KEGG) [19], Cluster of Orthologous Groups for eukaryotic complete genomes (KOG) [20], Translated EMBL-Bank (TrEMBL) [21] and NCBI non-redundant amino acid sequences (NR) [22] databases (Table S10).

Noncoding RNAs were also identified and annotated, including 208 microRNA genes (miRNAs), 73 rRNAs and 289 tRNAs. A total of 221 pseudogenes were identified in the *L. relictus* genome.

A total of 92.52 Mb of repeat sequence was annotated, composing 7.63% of the total genome length. We found that class I transposable elements (TEs) (RNA transposons or retrotransposons) occupied ~approximately 8.22% of the genome assembly. Among class I TEs, 1.12% were long terminal repeat elements (LTRs), 5.85% were long interspersed elements (LINEs) and 0.02% were short interspersed elements (SINEs) (Table S11). The LINE percentage from 4.95 to 6.03% and SINE percentage from 0.1 to 0.15% in five known species of Laridae genomes, respectively [17]. While the content of SINEs in *L. relictus* were obviously less common than in Laridae and this novel phenomenon needs to be futher studied. The *L. relictus* genome also contained class II TEs (DNA transposons), which occupied approximately 0.28% of the genome.

### Gene families

**Fig. 1:**
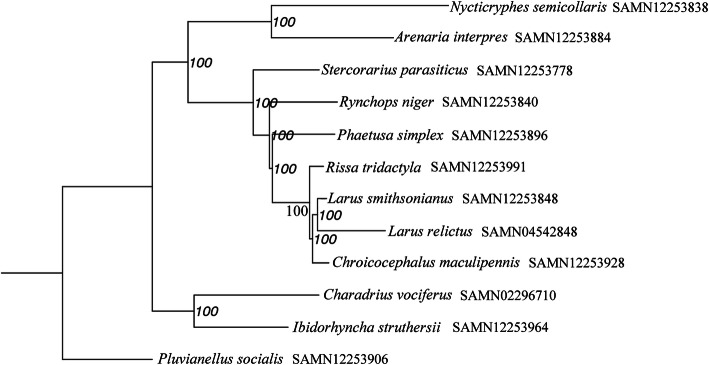
Topology of Maximum likelihood (ML) tree for 12 Charadriiformes species. Tree reconstruction based on single-copy orthologues protein sequences under IQ-TREE v1.6.11. BioSample numbers are indicated following species name. Numbers on nodes are bootstrap values

**Fig. 2:**
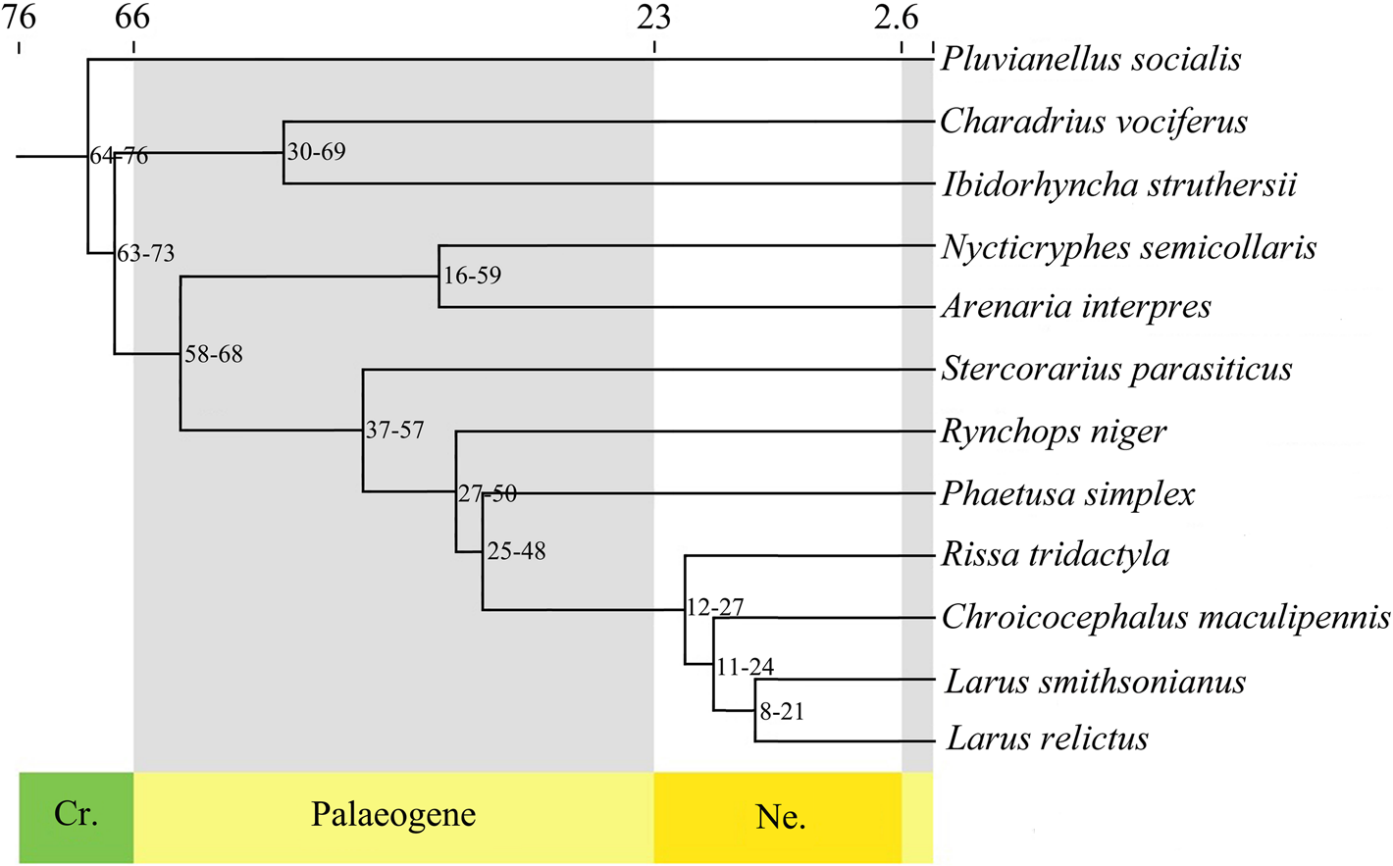
Timing of inferred divergence of 12 Charadriiformes species. Numbers on the nodes represent divergence times supported by 95% HPD

Comparison of the *L. relictus* genome assembly with the genomes of eleven other Charadriiformes species showed that a total of 14,453 genes of *L. relictus* could be clustered into 13,799 gene families, including 201 unique genes belonging to 62 gene families. The proportion of species-specific genes within *L. relictus* genome (1.1%) was obviously larger than that of other sampled genomes (0.0–0.1%) (Table S12). In addition, 5100 gene families were shared among all sampled species. The phylogenetic relationships based on 4902 single-copy orthologous genes indicated that all seven gulls were categorized into one branch, and *L. relictus* was genetically most related to another member of the order Laridae, *L. smithsonianus* in kinship (Fig. 1) with divergence time of 14.7 million years ago (Mya) (time 8–21 was supported by 95% highest posterior density (HPD) (Fig. 2).

### Positive selection genes and functional enrichment

We found that 842 single-copy orthologous genes were under positive selection in the *L. relictus* genome (Table S13). The GO annotation classifies the positively selected genes (PSGs) in terms of three categories: cellular component, biological process, and molecular function. Cellular component annotations were primarily cytosol and nuclear speck. Molecular functions were mainly ATP binding and chromatin binding. Biological process annotations were mainly positive regulation of transcription from RNA polymerase II promoter and ubiquitin-dependent protein catabolic process. In addition, we also identified the biochemical pathways of the PSGs. The KEGG annotation of the PSGs suggested that the pathway of RNA transport had the highest ratio, followed by spliceosome. (Fig. S2).

### Effective population size of *L. relictus*

**Fig. 3:**
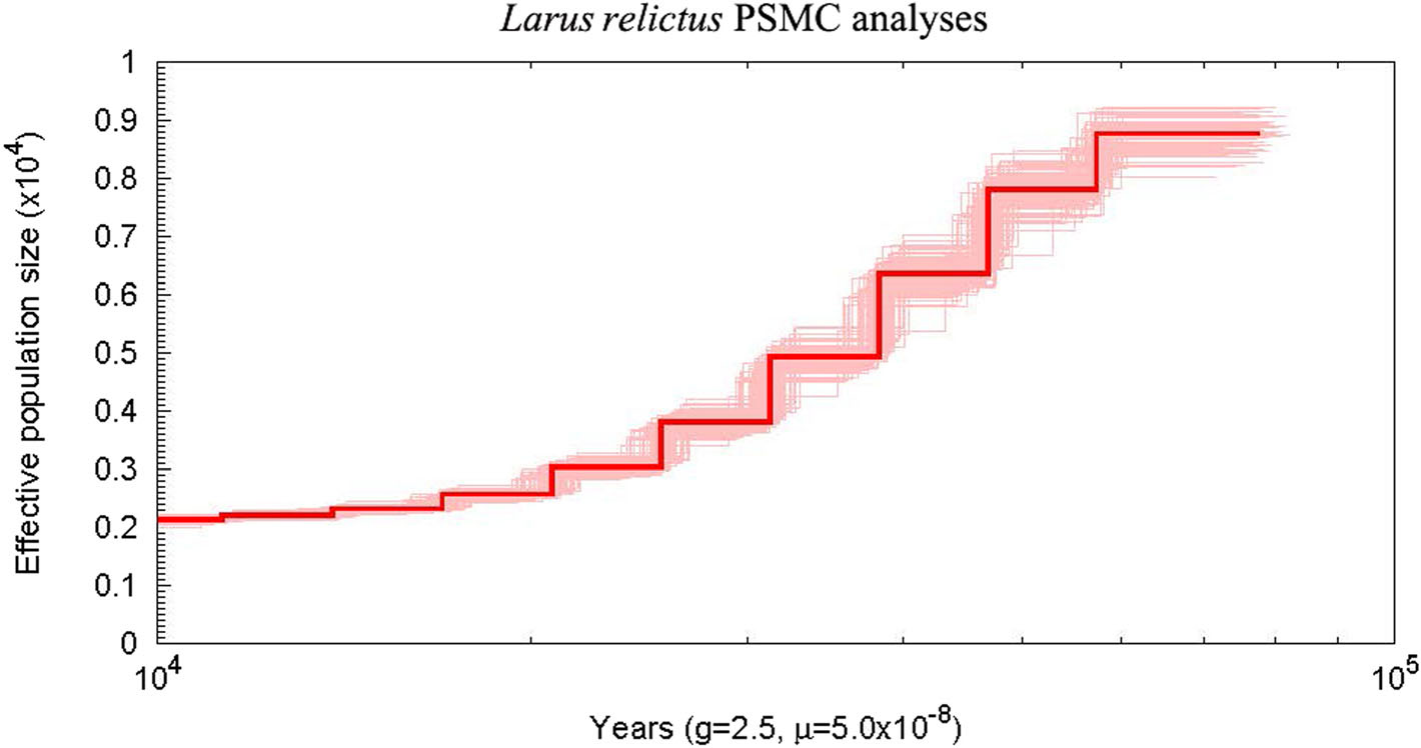
The PSMC analyses result of *Larus relictus*. An individual re-sequencing raw data was obtained in NCBI with accession number SRR14041273. One hundred iterations were performed

Pairwise sequentially Markovian coalescent (PSMC) analysis showed the demographic history of *L. relictus* from 100,000 years ago to 10,000 years ago. *L. relictus* had experienced a long period of population size decline, with the effective population size (Ne) from approximately 8800 individuals to 2200 individuals (Fig. 3).

## Discussion

### Genomic characteristics

The genome size of *L. relictus* was similar to those of five known species in Laridae, such as *L. smithsonianus* (1.20 Gb). The GC content of the *L. relictus* genome (43.11%) was higher than that of other known Laridae (42.28–42.95%) [17]. This proportion of repeat sequences is similar to that found in previous studies, in which almost all avian genomes contained lower levels of repeat elements than other animal genomes, with percentages of approximately 4.1–24.09%, except for the Red-headed Barbet (*Eubucco bourcierii*), with approximately 29.89% of its genome, the Coppersmith Barbet (*Psilopogon haemacephalus*) with 31.17%, and the Acacia Pied Barbet (*Tricholaema leucomelas*) with 31.47%, respectively [16, 17]. Genomes in different vertebrate lineages can have very different contents in repeate elements: the genomes of the primates contains more repeat elements (45–50% of the genome) than the genomes of mouse and rat (39–40%) and dog (34%) [23, 24].

#### Topological structure and evolution

Phylogenetic tree supported that Stercorariidae was so antiquated that it was divided out earlier than others in undergoing different selection pressures [25]. In *Larus*, *L. relictus* should be belonged to the Black-headed species, *L. smithsonianus* was belonged into White-headed species, but *Chroicocephalus maculipennis* was categorized into Masked species, respectively [26].

The timescale results indicated that the ancestral lineages of *L. relictus* and *L. smithsonianus* diverged approximately 14.7 Mya (Fig. 1). The genus *Larus* was split with *Rissa tridactyla* at approximately 20.51 Mya, which was close to that divergence time of the genus between *Larus* and *Rissa. Pluvianellus socialis* was divided out from other species were estimated at approximately 69.81 Mya, which is in agreement with the divergence time of the Charadriiformes as a whole (79–102 Mya) [27].

### Population dynamics

PSMC analyses revealed that *L. relictus* had took a long period of population size decline from 0.01-0.1 Mya, with very low effective population size 0.22 × 10^5^–0.88 × 10^5^ individuals (passenger pigeon, 1.3 × 10^5^–2.4 × 10^7^) [28]. Decrease in genetic diversity was reflected from this phenomenon, and consistent with previous studies (Pi, 0.00008–0.00041), then leaded the loss of many alleles in the population [2]. The average estimated expansion time of *L. relictus* was from 0.09 to 0.23 Mya, since the late to Middle Pleistocene (0.13–0.78 Mya) and early to Late Pleistocene (0.01–0.12 Mya) [2]. Synthetic analysis, recent range expansions following recovery from a bottleneck were determined between Middle Pleistocene and Late Pleistocene. The repeated glacial-interglacial changes during the Pleistocene period (0.01–1.9 Mya) might have influenced the expansion of *L. relictus*. Neverthelessly, we infered that the population size of *L. relictus* would be going a downward trend in the end of Late Pleistocene period and early Holocene.

## Conclusions

The whole-genome sequence of *L. relictus* was assembled employing the Illumina and PacBio sequencing platforms. The size of the final assembled genome was 1.21 Gb, with a contig N50 of 8.11 Mb and 92.52 (7.63%) Mb of repeat sequence, and 18,454 genes were predicted with 16,967 (91.94%) of these genes annotated.

Relict gull (*L. relictus*) has been holding a small effective population size and it has been experiencing very low genetic diversity and a long period of population decline while lacking a large geographical population. In this study, the genome information of *L. relictus* which is one of the few known reference genomes in genus *Larus,* will be effectively to investigate the evolutionary and molecular mechanisms of some significant processes in this species.

## Methods

### Sampling information

A naturally dead *L. relictus* fledgling from Hongjian Nur (39°04′ N, 109°53′ E), Yulin, Shaanxi Province, was collected and identified by H. Xiao, and the specimen (voucher number YG01) was deposited in the animal specimens museum of the Shaanxi Institute of Zoology, Xi’an, Shaanxi Province, China. Our team is a wildlife protection agency under the Shaanxi Academy of Sciences (China), cooperating and working with the authority department on Hongjian Nur for nearly 20 years, mainly devoted to the protection of the relict gull. To protect ***L. relictus***, this project has been approved and received permission from the Nature Reserve Authority of Hongjian Nur.

### DNA and RNA extraction

DNA was extracted from the muscle using the Cetyl Trimethyl Ammonium Bromide (CTAB) method, and total RNA was extracted from the heart, liver, spleen, lung and kidney of ***L. relictus*** using TRIzol reagent (Invitrogen, Carlsbad, CA, USA) following the protocol recommended by the manufacturer. DNA and RNA concentrations were measured using NanoDrop 2000, Qubit 2.0 and Agilent 2100. Only DNA with an DNA integrity number (DIN) and RNA with RNA integrity number (RIN) score > 8.0 and 1.8 < OD260/280 < 2.2 were used for the preparation and construction of PacBio and Illumina libraries.

### Library preparations (DNA and RNA) and sequencing

Both Illumina HiSeq 4000 and PacBio RSII sequencing platforms were used. For the Illumina pipeline, five short fragment paired-end libraries (three of 270 bp and two of 350 bp) were constructed using the standard Illumina protocol. The details of library construction are as follows: the genomic DNA was broken randomly using the ultrasonic method, and target fragments were filtered using magnetic beads for nucleic acid purification. The small fragment sequencing library was constructed through the steps of end repair, addition of polyA and adaptor, selection of target-size fragments and PCR.

For the long fragment libraries (three of 20 Kb) in the PacBio pipeline, the details of library construction are as follows: The genomic DNA was sheared using g-TUBE, followed by DNA damage-repair and end-repair. The dumbbell-type adapters were ligated, and exonuclease digestion was performed. BluePippin was used to select segments to obtain the sequencing library.

For the RNA fragment libraries (one of 280 bp and one of MicroRNA SE50) in the Illumina pipeline, the details of library construction are as follows: Briefly, rRNA was isolated from total RNA using Epicentre Ribo-Zero™ Kit and then fragmented randomly with Fragmentation Buffer. The first-strand cDNA was synthesized with random hexamer primers using the fragmented rRNA-depleted RNA as a template, and the second-strand cDNA was synthesized with DNA polymerase I (New England Biolabs) and RNase H (Invitrogen). After end repair, A-tail, adaptor ligation and purification with AMPure XP beads, PCR amplification was conducted.

The size and quality of all constructed libraries were evaluated using an Agilent 2100, NanoDrop 2000 and Qubit 2.0. Eligible libraries were sequenced on the Illumina HiSeq 4000 platform to generate 150 bp paired-end reads and PacBio RSII platform to generate Raw sequence data > 30.0GB. The Illumina HiSeq 4000 platform was also used for sequencing RNA data.

### Genome assembly assessment

Raw reads were filtered to remove adapter sequences (−e 0.1 -a AGATCGGAAGAGCACACGTCTGAACTCCAGTCAC -A AGATCGGAAGAGCGTCGTGTAGGGAAAGAGTGT -m 100 --cut 0 -O 3) and low-quality data (multi_rules, −u 0.1 -q 0.5 -w 10 -Q 33; Q20/30, −q 0.95/0.85 -w 30 -Q 33), with clean reads assembled using Trinity v2.4.0 [29]. After filtering out low-quality and less than 500 bp in length PacBio reads, LoRDEC v0.7 [30] software was used for error correction of PacBio data employing HiSeq data. The HiSeq data were preliminarily assembled by Platanus v1.2.4 [31] software. Using dbg2olc v4 [32] software, mixed assembly was carried out by using the data after error correction and the preliminary assembly results of HiSeq data. Pilon v1.22 [33] software was used to correct the assembly results using HiSeq data. To assess the completeness of the *L. relictus* genome assembly, we used two methods, with the first remapping the Illumina paired-end reads to the assembled genome and the second employing CEGMA v.2.5 [34] and BUSCO v3.0.2 databases.

### Genome annotation

Methods of ab initio-based, homologue-based and RNA-seq-based were used to predict gene structures, namely. EVM v1.1.1 [35] software was used to integrate the predicted genes and generate a consensus gene set. Then, GENSCAN v1.0 [36], Augustus v2.4 [37], GlimmerHMM v3.0.4 [38], GeneID v1.4 [39] and SNAP v4.0 [40] were first used to perform the ab initio prediction. For homologue prediction, GeMoMa v1.3.1 [41] was used, primarily employing five species as references, i.e., *G. gallus*, *Meleagris gallopavo*, *Taeniopygia guttata*, *Ficedula albicollis* and *Parus major*. Third, whole-transcriptomic data from the liver and an equal mix of five tissue RNA samples were used to assist genome annotations. HISAT v2.0.4 and StringTie v1.2.3 [42] were used for assembly based on RNA-seq reference data, and TransDecoder v5.0.1 [43] and GeneMarkS-T v5.1 [44] were applied to predict genes. PASA v2.0.2 [45] was used to predict unigene sequences assembled based on the whole transcriptome data without references. Finally, EVMv1.1.1 [35] was used to integrate the prediction results obtained by the above three methods, and PASA v2.0.2 [45] was used to predict alternative splice variants.

Software including LTR-FINDERv1.05 [46], MITE-Hunter v2011–11 [47], RepeatScout v1.05 [48] and PILER-DF v2.4 [49] was used for prediction of repetitive sequences in the *L. relictus* genome. A combination of structure-based and de novo strategies was used to construct repeat databases and then merged with Repbase [50] to form a final database. RepeatMasker v4.0.6 [51] was used to identify repeat sequences with this final repeat database.

Using the Rfam [52] and miRbase [53] databases as references, rRNA and microRNA were identified by Infernal v1.1 [54]. The tRNA was predicted using tRNAscan-SE v1.3.1 [55]. GenBlastA v1.0.4 [56] was used to search homologous gene sequences on the genome whose gene loci had been shielded. Pseudogenes were then identified via GeneWise v2.4.1 [57] with premature stop codons and frame shifts.

To assign gene functions in the *L. relictus* genome, we aligned the genes to five functional databases using BLASTv2.2.3 [58] (E-value = 1e-5). The databases included GO, KEGG, KOG, TrEMBL and NR.

### Phylogenetic analyses

We used the whole-genome sequence of *L. relictus* and 11 published whole-genome sequences of Charadriiformes species (*Arenaria interpres*, *Charadrius vociferous*, *Chroicocephalus maculipennis*, *Ibidorhyncha struthersii*, *L. smithsonianus*, *Nycticryphes semicollaris*, *Phaetusa simplex*, *P. socialis*, *R. tridactyla*, *Rynchops niger* and *Stercorarius parasiticus*). Orthofinder v2.4 (diamond, e = 0.001) was used to cluster gene families [59]. To assign gene functions of species-specific orthogroups, we aligned the genes to GO and KEGG functional databases using clusterProfile v3.14.0 [60].

A total of 4902 single-copy orthologues were identified, with protein sequences used for constructing phylogenetic trees. The protein sequences were aligned using MAFFT v7.205 (--localpair --maxiterate 1000) [61], with PAL2NAL v14 transferred protein alignment results into codon sequences [62]. Gblocks v0.91b (−b5 = h) [63] was used to remove the regions with poor alignments, and then concatenated into a combined dataset (super gene). ModelFinder was used to obtain the best model of GTR + F + I + G4 [64]. phylogenetic tree was constructed using the maximum likelihood (ML) algorithm with the JTT amino acid substitution model implemented in IQ-TREE v1.6.11 (bootstrap 1000) [65]. *P. socialis* was selected as outgroup.

Divergence times and ages of fossil records were derived from TimeTree (https://www.timetree.org/) and applied as the time control, i.e., 63.3–75.4 Mya of *P. socialis*-*S. parasiticus*, 59–80 Mya of *L. smithsonianus*-*N. semicollaris*, and 3.3–25.7 Mya of *L. smithsonianus*-*R. tridactyla*. Based on the results of phylogenetic tree, divergence time was estimated using the MCMCTree program in PAML v4.9i with model JC69 and correlated molecular clock. The consistency of the two repeated calculations was 1, and iteration parameters of a Markov chain: -burnin 5,000,000 -sampfreq 30 -nsample 5,000,000 [66]. MCMCTreeR v1.1 was used for graphical presentation.

In addition, the CodeML program in PAML v4.9i [66] included single-copy genes (F3x4 model of codon frequencies) was used to detect positively selected genes in the clade containing *L. relictus*, *L. smithsonianus*, *C. maculipennis*, *R. tridactyla* and *P. simplex*. Among them, the branch-site model was used, and likelihood ratio tests (LRTs) were calculated (*P* < 0.01) between Model A (foreground clade ω > 1) and null Model (any sites forbidden ω > 1). Posterior probability was calculated in Bayes empirical Bayes method (BEB).

### PSMC analyses

Consensus sequences of an individual re-sequencing (average depth: 29X; coverage ratio 10X: 92.44%) were called (SNP calling) using SAMtools v1.12, then converted into the fastq format using BCFtools v1.10 and Vcfutils (varFilter -D100 > var.flt.vcf). Bases of low sequencing depth (less than a third of the average depth) or high depth (twice the average depth) were masked. Sequences were split into short segments of 50 kb to estimate the demographic history with the Hidden Markov Model (HMM) model in PSMC v4.0.22 following parameters of -N25 -t15 -r5 -b -p (4 + 25 × 2 + 4 + 6) [67]. The generation time (g = 2.5) and mutation rates per year (u = 5 × 10^− 8^) were used. One hundred bootstraps were performed.

## Supplementary Information

**Additional file 1: Figure S1**. The results of gene prediction using three methods.

**Additional file 2: Figure S2**. The GO and KEGG annotation of PSGs. Only 10 items with the smallest *p*-value are shown.

**Additional file 3: Table S1.** Sequencing data by using Illumina platform. **Table S2** Raw data filtering by using PacBio platform. **Table S3** Statistics of subresds length distribution by using PacBio platform. **Table S4.** Statistics of genome assembly. **Table S5.** The mapped results using Illumina clean reads. **Table S6.** Statistics of genome assembly by using CEGMA v2.5. **Table S7.** Genome completeness assessment employing BUSCO v3.0.2. **Table S8.** Statistics of gene prediction. **Table S9.** Statistics of gene information from 10 species. **Table S10.** Statistic information of gene function annotation. **Table S11.** Repeat elements in the genome. **Table S12.** Classification and statistics of gene families.

**Additional file 4: Table S13.** Statistics of *Larus relictus* positively selected genes.

## Data Availability

The authors declare that the data supporting the finding of this study are available in the article and its supplementary information files. The raw sequencing reads data were deposited to NCBI as part of the BioProject PRJNA314730 via Sequence Read Archive (SRA). PacBio DNA-seq, Illumina DNA-seq, RNA-seq and Illumina DNA re-seq were available in SRR12874010, SRR12874011, SRR12874012, SRR12874013, SRR14041273, respectively.

## Abbreviations

PacBio: Pacific Biosciences
CEG: Core Eukaryotic Gene
GO: Gene Ontology
KEGG: Kyoto Encyclopedia of Genes and Genomes
KOG: Cluster of Orthologous Groups for eukaryotic complete genomes
TrEMBL: Translated EMBL-Bank
NR: NCBI non-redundant amino acid sequences
miRNAs: microRNA genes
TEs: Transposable Elements
LINEs: Long Interspersed Elements
LTRs: Long Terminal Repeats
SINEs: Short Interspersed Elements
Mya: Million years ago
HPD: Highest Posterior Density
PSGs: Positively Selected Genes
PSMC: Pairwise Sequentially Markovian Coalescent
*CTAB*: Cetyl Trimethyl Ammonium Bromide
DIN: DNA Integrity Number
RIN: RNA Integrity Number
LRTs: Likelihood Ratio Tests
BEB: Bayes Empirical Bayes method
