# Supplementary figures and images for: Genome-wide analyses of the relict gull (Larus relictus): insights and evolutionary implications
Source: BMC Genomics. 2021 Apr 29;22:311. doi: 10.1186/s12864-021-07616-z (PMC8082828; doi:10.1186/s12864-021-07616-z)

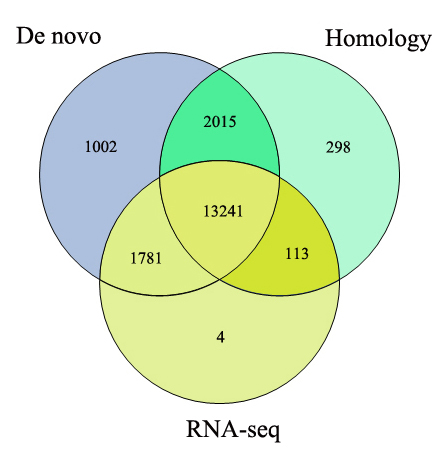

Supplement: Supplementary file 1 — Additional file 1: Figure S1. The results of gene prediction using three methods. [file 12864_2021_7616_MOESM1_ESM.jpg]

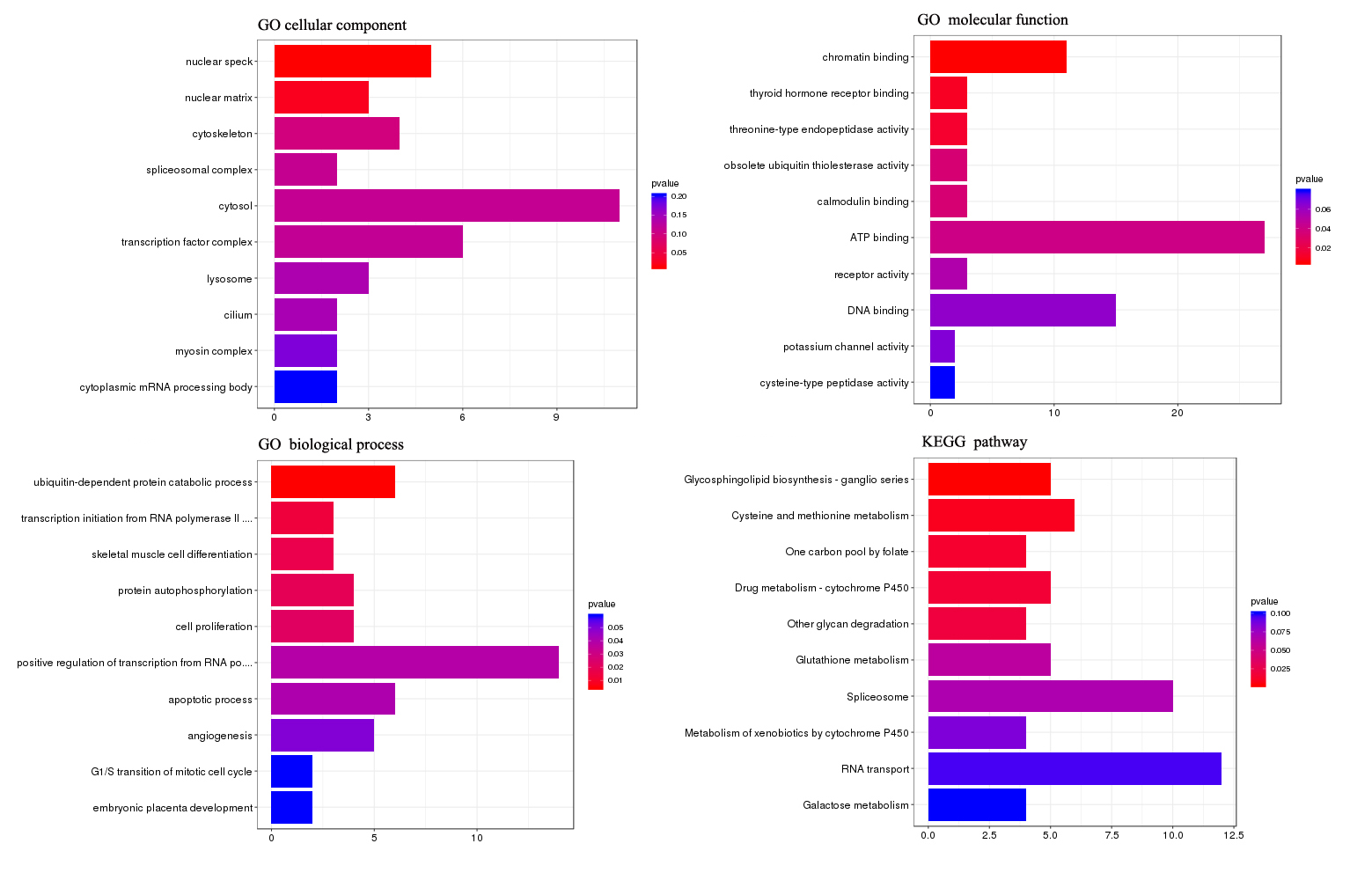

Supplement: Supplementary file 2 — Additional file 2: Figure S2. The GO and KEGG annotation of PSGs. Only 10 items with the smallest p-value are shown. [file 12864_2021_7616_MOESM2_ESM.jpg]
