# Supplementary material for: Genome-wide analyses of the relict gull (Larus relictus): insights and evolutionary implications
Source: BMC Genomics. 2021 Apr 29;22:311. doi: 10.1186/s12864-021-07616-z (PMC8082828; doi:10.1186/s12864-021-07616-z)
Supplement: Supplementary file 3 — Additional file 3: Table S1. Sequencing data by using Illumina platform. Table S2 Raw data filtering by using PacBio platform. Table S3 Statistics of subresds length distribution by using PacBio platform. Table S4. Statistics of genome assembly. Table S5. The mapped results using Illumina clean reads. Table S6. Statistics of genome assembly by using CEGMA v2.5. Table S7. Genome completeness assessment employing BUSCO v3.0.2. Table S8. Statistics of gene prediction. Table S9. Statistics of gene information from 10 species. Table S10. Statistic information of gene function annotation. Table S11. Repeat elements in the genome. Table S12. Classification and statistics of gene families. [file 12864_2021_7616_MOESM3_ESM.docx]

**Table S1** Sequencing data by using Illumina platform.

| Library | Data (Gb) | Sequencing depth (×) | Q20 (%) | Q30(%) |
| --- | --- | --- | --- | --- |
| 270 bp_1 | 19.89 | 16.44 | 96.24 | 91.44 |
| 270 bp_2 | 25.01 | 20.67 | 96.06 | 91.14 |
| 270 bp_3 | 23.37 | 19.31 | 95.97 | 90.90 |
| 350 bp_1 | 20.93 | 17.30 | 95.60 | 90.13 |
| 350 bp_2 | 17.10 | 14.13 | 95.58 | 90.06 |
| Total | 106.29 | 87.85 | --- | --- |

**Table S2** Raw data filtering by using PacBio platform.

| **Library** | **Reads number** | **Total_base (bp)** | **Read_N50** | **Mean reads length** | **Maximum reads length** |
| --- | --- | --- | --- | --- | --- |
| 20 Kb | 3,623,141 | 30,500,420,341 | 12,712 | 8,418 | 104,462 |

**Table S3** Statistics of subresds length distribution by using PacBio platform.

| **Length (bp)** | **Number** | **Total Length (bp)** | **Average length (bp)** |
| --- | --- | --- | --- |
| 0~2000 | 552,056 | 667,098,419 | 1,208 |
| 2000~4000 | 567,476 | 1,682,161,740 | 2,964 |
| 4000~6000 | 477,063 | 2,373,103,193 | 4,974 |
| 6000~8000 | 406,652 | 2,834,808,752 | 6,971 |
| 8000~10000 | 341,606 | 3,064,140,746 | 8,970 |
| 10000~12000 | 303,332 | 3,333,660,543 | 10,990 |
| 12000~14000 | 285,619 | 3,708,049,972 | 12,983 |
| 14000~16000 | 230,500 | 3,444,672,311 | 14,944 |
| 16000~18000 | 158,350 | 2,681,002,292 | 16,931 |
| 18000~ | 300,487 | 6,711,722,373 | 22,336 |
| Total | 3,623,141 | 30,500,420,341 | 8,418 |

**Table S4** Statistics of genome assembly.

| Contig number | Contig length (bp) | Contig N50 (bp) | Contig N90 (bp) | Contig max (bp) | GC content (%) | Gap total length (bp) |
| --- | --- | --- | --- | --- | --- | --- |
| 1,313 | 1,211,971,565 | 8,107,927 | 1,799,621 | 29,690,937 | 43.11 | 0 |

Contigs length are all greater than 1kb.

**Table S5** The mapped results using Illumina clean reads.

| Library | Total reads | Mapped (%) | Properly mapped (%) |
| --- | --- | --- | --- |
| 270 bp_1 | 119,658,048 | 99.97% | 93.77% |
| 270 bp_2 | 150,406,746 | 99.96% | 93.44% |
| 270 bp_3 | 140,027,108 | 99.97% | 93.33% |
| 350 bp_1 | 124,560,314 | 99.97% | 93.71% |
| 350 bp_2 | 101,671,966 | 99.97% | 93.70% |

**Table S6** Statistics of genome assembly by using CEGMA v2.5.

| Species | Number of 458 CEGs* present in assembly | % of 458 CEGs present in assemblies | Number of 248 highly conserved CEGs present | % of 248 highly conserved CEGs present |
| --- | --- | --- | --- | --- |
| *Larus relictus* | 416 | 90.83% | 175 | 70.56% |

* represents Core Eukaryotic Genes

**Table S7** Genome completeness assessment employing BUSCO V3.0.2.

| BUSCO benchmark | Gene number | Percentage (%) |
| --- | --- | --- |
| Total BUSCO groups searched | 4,915 | - |
| Complete BUSCOs (C) | 4,555 | 92.7 |
| Complete and single-copy BUSCOs (S) | 4,507 | 91.7 |
| Complete and duplicated BUSCOs (D) | 48 | 1.0 |
| Fragmented BUSCOs (F) | 212 | 4.3 |
| Missing BUSCOs (M) | 148 | 3.0 |

**Table S8** Statistics of gene prediction

| **Prediction strategy** | **Software** | **Homologue-based** **species** | **Gene number** |
| --- | --- | --- | --- |
| *Ab initio* | Genscan v1.0 | - | 46,679 |
|  | Augustus v2.4 | - | 25,055 |
|  | GlimmerHMM v3.0.4 | - | 91,551 |
|  | GeneID v1.4 | - | 40,016 |
|  | SNAP v4.0 | - | 68,812 |
| Homology-based | GeMoMa v1.3.1 | *Gallus gallus* | 11,613 |
|  |  | *Meleagris gallopavo* | 16,910 |
|  |  | *Taeniopygia guttata* | 14,276 |
|  |  | *Ficedula albicollis* | 15,140 |
|  |  | *Parus major* | 15,678 |
| RNAseq | PASA v2.0.2 | - | 24,412 |
|  | TransDecoder v2.0 | - | 42,086 |
|  | GeneMarkS-T v5.1 | - | 25,601 |
| Integration | EVM v1.1.1 | - | 18,454 |

**Table S9** Statistics of gene information from ten species.

| Species | Gene number | Average gene  lengh (bp) | Average exon  lengh (bp) | Average intron  lengh (bp) | Average CDS*  lengh (bp) |
| --- | --- | --- | --- | --- | --- |
| *Larus relictus* | 18,454 | 20,882 | 165 | 1,997 | 1,577 |
| *Larus smithsonianus* | 15,503 | 11,215 | 178 | 1,823 | 1,161 |
| *Phaetusa simplex* | 15,243 | 17,350 | 172 | 2,345 | 1,350 |
| *Rissa tridactyla* | 16,255 | 19,499 | 171 | 2,522 | 1,398 |
| *Rynchops niger* | 16,112 | 18,463 | 166 | 2,359 | 1,369 |
| *Chroicocephalus maculipennis* | 15,643 | 16,464 | 173 | 2,305 | 1,315 |
| *Charadrius vociferus* | 16,856 | 19,100 | 162 | 2,482 | 1,324 |
| *Gallus gallus* | 16,516 | 21,100 | 158 | 2,437 | 1,433 |
| *Homo aspiens* | 20,424 | 48,900 | 174 | 5,500 | 1,663 |
| *Mus musculus* | 23,038 | 35,100 | 179 | 4,429 | 1,538 |

* represents Coding sequence

**Table S10** Statistic information of gene function annotation.

| Annotation database | Annotated gene number | Percentage (%)* |
| --- | --- | --- |
| GO | 5,324 | 28.85% |
| KEGG | 10,543 | 57.13% |
| KOG | 12,036 | 65.22% |
| TrEMBL | 16,857 | 91.35% |
| NR | 16,957 | 91.89% |
| All | 16,967 | 91.94% |

* represents percentage of the total number of genes annotated to the database

**Table S11** Repeat elements in the genome.

| Repeated sequence  type | Repeated sequence number | Repeated sequence  Length (bp) | Percentage (%)* |
| --- | --- | --- | --- |
| ClassI/DIRS | 1,028 | 202,447 | 0.02 |
| ClassI/LINE | 203,699 | 70,859,111 | 5.85 |
| ClassI/LTR | 31,153 | 13,226,525 | 1.09 |
| ClassI/LTR/Copia | 361 | 20,464 | 0 |
| ClassI/LTR/Gypsy | 4,661 | 415,711 | 0.03 |
| ClassI/PLE\|LARD | 51,486 | 140,473,81 | 1.16 |
| ClassI/SINE | 1,666 | 251,526 | 0.02 |
| ClassI/TRIM | 657 | 579,390 | 0.05 |
| ClassI/Unknown | 346 | 30,090 | 0 |
| ClassII/Crypton | 268 | 15,586 | 0 |
| ClassII/Helitron | 1,193 | 109,408 | 0.01 |
| ClassII/MITE | 24 | 5,542 | 0 |
| ClassII/Maverick | 1,736 | 436,382 | 0.04 |
| ClassII/TIR | 16,821 | 2,227,932 | 0.18 |
| ClassII/Unknown | 7,053 | 610,934 | 0.05 |
| Potential Host Gene | 553 | 169,124 | 0.01 |
| SSR | 498 | 77,508 | 0.01 |
| Unknown | 13,738 | 4,021,273 | 0.33 |
| Total without overlap | 336,941 | 92,521,240 | 7.63 |

* represents the proportion of repeated sequences in the genome.


**Table S12** Classification and statistics of gene families.

| ***Item*** | ***Arenaria interpres*** | ***Chroicocephalus maculipennis*** | ***Rynchops niger*** | ***Stercorarius parasiticus*** | ***Larus***  ***relictus*** | ***Nycticryphes semicollaris*** | ***Phaetusa simplex*** | ***Larus smithsonianus*** | ***Pluvianellus socialis*** | ***Ibidorhyncha struthersii*** | ***Rissa tridactyla*** | ***Charadrius vociferus*** |
| --- | --- | --- | --- | --- | --- | --- | --- | --- | --- | --- | --- | --- |
| **Number of genes** | 13872 | 13945 | 14332 | 13876 | 18423 | 14324 | 13260 | 14080 | 14317 | 13853 | 14776 | 13918 |
| **Number of genes**  **in orthogroups** | 13678 | 13857 | 14206 | 13777 | 14453 | 14176 | 13141 | 13748 | 13996 | 13705 | 14682 | 13100 |
| **Number of**  **unassigned genes** | 194 | 88 | 126 | 99 | **3970** | 148 | 119 | 332 | 321 | 148 | 94 | 818 |
| **Percentage of**  **genes in orthogroups** | 98.6 | 99.4 | 99.1 | 99.3 | 78.5 | 99.0 | 99.1 | 97.6 | 97.8 | 98.9 | 99.4 | 94.1 |
| **Percentage of**  **unassigned genes** | 1.4 | 0.6 | 0.9 | 0.7 | 21.5 | 1.0 | 0.9 | 2.4 | 2.2 | 1.1 | 0.6 | 5.9 |
| **Number of orthogroups containing species** | 13374 | 13536 | 13915 | 13484 | 13799 | 13860 | 12865 | 13426 | 13744 | 13383 | 14447 | 12748 |
| **Percentage of**  **orthogroups**  **containing species** | 65.8 | 66.6 | 68.5 | 66.4 | 67.9 | 68.2 | 63.3 | 66.1 | 67.6 | 65.9 | 71.1 | 62.7 |
| **Number of**  **species-specific orthogroups** | 5 | 1 | 2 | 1 | **62** | 1 | 0 | 6 | 3 | 2 | 2 | 8 |
| **Number of genes in**  **species-specific orthogroups** | 10 | 2 | 4 | 2 | **201** | 2 | 0 | 13 | 7 | 4 | 4 | 17 |
| **Percentage of genes in species-specific orthogroups** | 0.1 | 0.0 | 0.0 | 0.0 | 1.1 | 0.0 | 0.00 | 0.1 | 0.0 | 0.0 | 0.0 | 0.1 |

Genes with frameshift mutations in all species were excluded from the statistics. Total orthogroups number: 20318.

**Table S13.** Statistics of *Larus relictus* positively selected genes. * represents a significance greater than 0.95. ** represents extremely significant, with significance greater than 0.99.
